# The Effect of Taurine on the Alkalinity Stress Resistance of *Eriocheir sinensis*

**DOI:** 10.3390/ani16172749

**Published:** 2026-09-02

**Authors:** Jingyao Wang, Haoyang Sheng, Changhao Shao, Bohao Wang, Shengqiang Tao

**Affiliations:** 1 Office of Academic Affairs, Beihua University, Jilin 132013, China; 2 College of Animal Science and Technology, Northeast Agricultural University, Harbin 150030, China; 3 Tianjin Tiancheng Pharmaceutical Safety Evaluation Co., Ltd., Tianjin 300000, China

**Keywords:** alkalinity stress, taurine, *Eriocheir sinensis*, apoptosis

## Abstract

High alkalinity, a major stress in saline–alkaline water, often leads to oxidative damage and apoptosis. Taurine has been shown to enhance the tolerance of aquatic animals to environmental stress, but whether it can reduce toxicity of alkalinity on Chinese mitten crab (*Eriocheir sinensis*) has not been determined. Thus, in this study, we investigated whether taurine could alleviate the deleterious effects of alkalinity stress on *E. sinensis*. The results revealed that taurine can improve the survival rate of the crab under 35.00 mmol/L alkalinity stress. Meanwhile, taurine inhibited the production of reactive oxygen species (ROS) and ameliorated the oxidative damage induced by alkalinity stress in crab. Taurine also inhibited alkalinity stress-mediated induction of mitogen-activated protein kinase-related pathways, subsequently suppressing the mRNA expression of *Caspase 8* and *Bax* and preventing the occurrence of alkalinity stress-induced apoptosis in *E. sinensis*. In addition, inhibition of alkalinity stress-induced apoptosis by taurine may be related to the ability of taurine to bind to MAPK proteins. Overall, these results suggested that taurine can relieve the toxicity of high alkalinity on *E. sinensis* through mitigating oxidative damage and inhibiting apoptosis, supporting its applicability as a treatment for oxidative damage induced by high alkalinity.

## Simple Summary

High alkalinity in saline–alkaline water is a major problem for aquaculture, causing oxidative stress and cell death in aquatic animals. This study tested whether taurine, a common feed additive, could protect Chinese mitten crabs from such stress. We found that taurine significantly improved the survival rate of crabs exposed to high alkalinity. It reduced harmful reactive oxygen species, lessened oxidative damage, and prevented cells from undergoing programmed death. Taurine worked by suppressing specific stress-related signaling pathways inside cells. Our results show that taurine can effectively counteract the toxic effects of alkalinity, offering a practical nutritional strategy to improve crab health and productivity in saline–alkaline waters. This finding may help farmers raise crabs more successfully in areas with poor water quality, supporting sustainable aquaculture development.

## 1. Introduction

In recent years, saline–alkaline water aquaculture has become an important research direction in the field of aquaculture [1,2,3]. Carbonate-type saline–alkaline water has been documented to exert adverse impacts on the growth performance and physiological health of aquatic animals [4,5], ultimately resulting in severely suppressed aquaculture yields [6,7]. The toxic effects triggered by alkalinity are mainly mediated by synchronous increases in water pH and bicarbonate HCO_3_^−^ concentrations. Combined high pH and HCO_3_^−^ concentrations trigger exfoliation, rupture and hyperplasia of gill epithelia cells, thereby obstructing branchial ion transport; excess bicarbonate further disturbs blood CO_2_ homeostasis and gives rise to systemic toxic stress [8]. In addition, elevated alkalinity drives massive accumulation of reactive oxygen species [9,10], which breaks down the host’s antioxidant defense system and sequentially initiates cellular apoptosis and inflammatory immune cascades [11]. Previous research on black sea bream (*Acanthopagrus schlegelii*) demonstrated that chronic exposure to 30 mmol/L NaHCO_3_ can induce progressive hepatic damage, oxidative stress, inflammatory response and metabolic disorder [12]. To mitigate the detrimental impacts of alkaline stress on aquatic animals, nutritional intervention has been extensively explored as a promising strategy to enhance the stress resistance and production performance of aquatic animals farmed in saline–alkaline water. Fan et al. [13] reported that optimizing dietary protein levels could effectively relieve oxidative damage, intestinal inflammatory responses and growth suppression in common carp (*Cyprinus carpio*) exposed to prolonged alkalinity stress. Likewise, dietary astaxanthin supplementation was verified to suppress mitochondrial apoptosis triggered by high alkaline stress in *Exopalaemon carinicauda* [14,15]. However, the efficacy of nutritional modulation in alleviating saline–alkaline water responses in aquatic animals remains insufficiently investigated.

Taurine as a feed additive has been extensively studied and shown to improve stress resistance through various pathways. For example, taurine scavenges excess free radicals to attenuate oxidative cellular damage and alleviate ammonia toxic effects [16]. Furthermore, taurine also plays critical protective roles under low temperature and heavy metal exposure, mainly by boosting the host’s antioxidant capacity and suppressing cellular apoptosis [17,18]. Mitogen-activated protein kinase (MAPK) acts as a critical signaling mediator that relays extracellular signals to the nucleus, and its activation triggered by accumulated reactive oxygen species (ROS) further initiates cellular apoptosis [19]. Our previous studies revealed that alkalinity stress disrupt ammonia metabolism and activates the ROS/MAPK signaling pathway to trigger cellular apoptosis, which ultimately reduces the survival rate of crabs [4]. Nevertheless, it remains elusive whether taurine exerts beneficial effects against alkalinity-induced oxidative stress and apoptosis via modulation of the MAPK signaling pathway.

Chinese mitten crab (*Eriocheir sinensis*) is an economically important crustacean in China, maintaining an annual production of about 800,000 tons due to its delicious taste and rich nutritional content [20]. In recent years, it has been used as one of the main crab species cultured in carbonate alkaline water owing to its adaptability to alkalinity. However, high alkalinity in the water often results in slow growth of river crabs, leading to reduced productivity. Previous studies have confirmed that high alkalinity triggers oxidative stress in the hepatopancreas, which further causes hepatopancreatic structural damage [21,22,23], gill cell apoptosis [4] and an increased mortality rate [8]. Therefore, it is essential to research methods to enhance the growth performance of *Eriocheir sinensis* in high carbonate alkaline water. Because taurine possesses various biological functions, the administration of taurine may potentially mitigate the adverse effects of alkalinity stress on crabs. Thus, the present study investigated whether taurine could mitigate the adverse effects of alkalinity stress on crabs by regulating serum ammonia levels, antioxidant capacity and cellular apoptosis; furthermore, we aimed to reveal the underlying regulatory mechanism. The study can partially reveal the physiological responses and molecular mechanisms of taurine’s protective effect against alkalinity stress in *E. sinensis*, providing guidance and a theoretical basis for the development of feed for aquaculture in saline–alkaline water.

## 2. Materials and Methods

### 2.1. Crabs

A total of 1500 healthy crabs used in the present study were purchased from a breeding farm located in Panshan County, Panjin City, Liaoning Province, China. The water alkalinity at the farm was approximately 2.4 mmol/L, with a salinity of around 1‰. The average water temperature in the ponds during the growth period of juvenile Chinese mitten crabs was approximately 20 °C. Prior to the commencement of the formal experiment, the crabs were acclimated in 50 glass tanks (1.0 m × 0.5 m × 0.6 m, water depth about 0.3 m) and fed a commercial diet for 2 weeks to mitigate transport-induced stress and acclimate to experimental conditions. During the acclimation period, the water temperature was maintained at 24 ± 1 °C using a liquid-in-glass thermometer, the pH was maintained at 7.2 with a Leici PHS-25 pH meter, and dissolved oxygen levels were kept above 6 mg/L as measured by a Yukaixin portable dissolved oxygen detector. In the present study, all crabs received proper care following the guidelines outlined in the Care and Use of Laboratory Animals in China.

### 2.2. Experiment Design

(1) The effect of taurine on survival rate of *E. sinensis* exposed to high alkalinity stress

To investigate whether taurine can mitigate the detrimental effects of alkalinity on the crab, the experiment firstly examined the impact of different taurine levels on the survival rate of crab under 35.00 mmol/L alkalinity stress for 96 h. Analytically pure sodium bicarbonate was used to adjust the alkalinity of water, and the alkalinity was determined by the methyl orange hydrochloric acid calibration method. The water in the experiment was changed every 24 h to maintain a stable alkaline environment. A total of 225 crabs (average weight: 12.32 ± 0.56 g) were divided into 5 groups, each containing 3 replicates of 15 crabs. The control group was cultured in freshwater and injected with 0.15 mL of physiological saline, while four experimental groups maintained at 35.00 mmol/L alkalinity received intraperitoneal injections of 0.15 mL of taurine solution at doses of 0, 2.4, 9.6 and 96 mg/mL per 12 g of body weight, respectively. The groups were designated as follows: T0, T0/CA, T1/CA, T2/CA, and T3/CA, respectively. During the 96 h period of stress, water alkalinity was maintained at 35.00 mmol/L, water temperature at 24 ± 1 °C, pH at 8.9, and dissolved oxygen above 6 mg/L. The number of dead crabs in each group was recorded daily, and mortalities were promptly removed from the tanks.

(2) The effect of taurine on *E. sinensis* exposed to sublethal alkalinity stress

To further investigate the mechanism of taurine in alleviating alkalinity stress in crabs, the 1/4 96 h lethal concentration (17.50 mmol/L alkalinity) was selected to stress the crab [8], and the alleviating dosage of taurine was chosen as 0.15 mL of taurine solution at a concentration of 96 mg/mL per 12 g of body weight. The study included three experimental groups: the control group (received 0.15 mL of physiological saline per 12 g of body weight), the CA group (received 0.15 mL of physiological saline per 12 g of body weight with an alkalinity of 17.50 mmol/L in the aquarium), and the CA + T group (received 0.15 mL of a 96 mg/mL taurine solution per 12 g of body weight, also with an alkalinity of 17.50 mmol/L in the aquarium). Each treatment group included three biological replicates of 12 crabs (average weight: 12.52 ± 0.28 g), and all crabs were reared in 1.0 m × 0.5 m × 0.6 m glass tanks with a constant water depth of approximately 0.3 m. During the 96 h period of stress, the water temperature was maintained at 24 ± 1 °C, with ammonia nitrogen and nitrite levels consistently below 0.1 mg/L and 0.05 mg/L, respectively. The pH levels in the control group, CA group and CA + T group were measured as 7.2 ± 0.1, 8.7 ± 0.1 and 8.7 ± 0.1, respectively. Continuous aeration was provided to each culture tank to ensure an adequate oxygen supply, maintaining dissolved oxygen concentration above 6 mg/L. The water in the experiment was changed every 24 h to maintain a stable alkaline environment. The crabs were not fed during the experiment and were exposed to the natural light cycle.

### 2.3. Sample Collection

After 96 h of alkalinity stress, six crabs were randomly selected from each aquarium, and they were anesthetized using crushed ice. Subsequently, hemolymph was carefully extracted from the base of the appendages using 1 mL syringes and collected in 1.5 mL centrifuge tubes. After refrigerating at 4 °C for 4 h, the samples were crushed and centrifuged at 7000 rpm for 10 min. The supernatant obtained from centrifugation was carefully transferred to new centrifuge tubes and stored in a −80 °C freezer. Using surgical scissors and a scalpel, gill tissues were rapidly dissected, frozen in liquid nitrogen, and stored in a −80 °C freezer. To ensure an adequate sample size of hemolymph and gill tissue for measuring the parameters, a strategy of combining samples was adopted. Specifically, every two samples from the same aquarium were combined into one sample. In addition, three more crabs were randomly selected from each aquarium to obtain hemolymph samples, which were then placed into 1.5 mL centrifuge tubes containing an anticoagulant solution (hemolymph: anticoagulant = 1:1) for the detection of reactive oxygen species activity and cellular apoptosis. The anticoagulant solution comprised glucose, trisodium citrate dihydrate, sodium chloride, and EDTA-Na2, with concentrations of 0.1 mol/L, 20 mmol/L, 26 mmol/L, 0.14 mol/L, and 10 mmol/L, respectively.

### 2.4. Physiological and Biochemical Parameters

Ammonia levels (blood ammonia assay kit, A086-1-1), urea nitrogen (urea assay kit, C013-2-1) levels, glutamine synthetase activity (glutamine synthetase assay kit, A047-1-1), triglyceride (triglyceride assay kit, A110-1-1), total cholesterol (total cholesterol assay kit, A111-1-1) and glucose (glucose assay kit, F006-1-1) in hemolymph were determined using commercial assay kits (Nanjing Jiancheng Bioengineering Institute, Nanjing, China).

### 2.5. Antioxidant Parameters

The detection of antioxidant enzymes primarily utilized the following assay kits: the WST-1 method for determining superoxide dismutase activity (SOD), the ammonium molybdate method for measuring catalase activity (CAT), the ABTS method for assessing total antioxidant capacity activity (T-AOC), and the TBA method for quantifying malondialdehyde content (MDA). The protein content in the gills was determined using the Coomassie brilliant blue method. These assay kits are all sourced from Nanjing Jiancheng Bioengineering Institute, Nanjing, China.

### 2.6. Reactive Oxygen Species Content

The 1 mL hemolymph samples containing anticoagulant were centrifuged at 4 °C, 1000× *g* for 5 min. The collected cells were gently washed twice with phosphate-buffered saline (PBS, pH 7.4) and then mixed with a 1‰ solution of 2′,7′-dichlorodihydrofluorescein diacetate (DCFH-DA) fluorescent probe. The mixture was then incubated at 37 °C for 60 min on a shaker. After incubation, the cells were gently washed twice with pH 7.4 PBS to remove any residual probe. Subsequently, the level of ROS in the hemolymph was detected using a NovoCyte flow cytometer (Agilent Technologies (China) Co., Ltd., Hangzhou, China) with excitation at a wavelength of 488 nm. The ROS assay kit used in the experiment was provided by Jiangsu Jiancheng Bioengineering Institute (reactive oxygen species assay kit, E004-1-1).

### 2.7. Apoptosis Flow-Cytometry Assay

The hemolymph samples containing anticoagulant were centrifuged at 4 °C, 800× *g* for 5 min. The collected cells were washed twice with pH 7.4 PBS and then the cell concentration was adjusted to 1–5 × 10^5^. Subsequently, 0.5 mL of binding buffer was added to suspend the cells. After that, 5 uL of Annexin V-FITC was added and mixed well, followed by the addition of 5 uL of propidium iodide and thorough mixing. Additionally, control groups of cells required treatments with no dye, single staining with Annexin-V-FITC, and single staining with propidium iodide for fluorescence compensation of the experimental samples. The mixture was then incubated at room temperature in the dark for 10 min before being subjected to detection using a NovoCyte flow cytometer.

### 2.8. RT-qPCR Analysis

Total RNA was extracted from the gills using Trizol according to the manufacturer’s instructions (RR820A, Takara, Dalian, China), and cDNA synthesis was performed using the PrimeScript RT Reagent Kit. The concentration and quality of RNA were assessed using a NanoDrop spectrophotometer (Implen GmbH, Munich, Germany) at 260/280 nm. The concentration of cDNA for RT-qPCR was adjusted to 1000 ng/μL. The primers used in this study were designed based on the research of Wang et al. [24]. The primer sequences are shown in Table 1. β-actin and S27 were used as reference genes for normalization of gene expression. Real-time PCR was performed on an ABI 7500 real-time instrument (Applied Biosystems, Foster City, CA, USA) using the TB Green Premix Ex Taq II kit according to the manufacturer’s instructions. The real-time PCR amplification conditions included an initial denaturation step at 94 °C for 30 s, followed by 40 cycles of denaturation at 94 °C for 5 s and annealing/extension at 60 °C for 30 s. The relative gene expression was calculated using the 2^−ΔΔCT^ method [25].

### 2.9. Molecular Docking

Molecular docking was applied to predict the likely binding modes of a ligand (taurine) with MAPK protein. Briefly, the 3D structure for MAPK target was built from SWISS-MODEL (https://swissmodel.expasy.org (accessed on 5 June 2026)), and the 3D conformer for ligand was downloaded from PubChem (https://pubchem.ncbi.nlm.nih.gov (accessed on 5 June 2026)). For ligand–protein docking, AutoDock 4.2.6 software removed water molecules, indicated hydrogens and discarded any structures that lacked the biologically necessary cofactors. Then, PyMol 2.6.0 found the most favorable binding mode of taurine to MAPK; this was outputted after optimization.

### 2.10. Statistical Analysis

The statistical analysis was conducted using SPSS 19.0 software. Data are presented as mean ± standard deviation (SD). Levene’s test was used to assess the homogeneity of variance. In cases where the data did not meet the assumption of homogeneity of variance, Dunnett’s T3 test was used for analysis. For data that met the assumption of homogeneity of variance, one-way ANOVA was performed to analyze the experimental results, followed by Duncan’s multiple comparison test. A significance level of *p* < 0.05 was considered statistically significant.

## 3. Results

### 3.1. Survival Rate Under 35.00 mmol/L Alkalinity Stress

**Figure 1:**
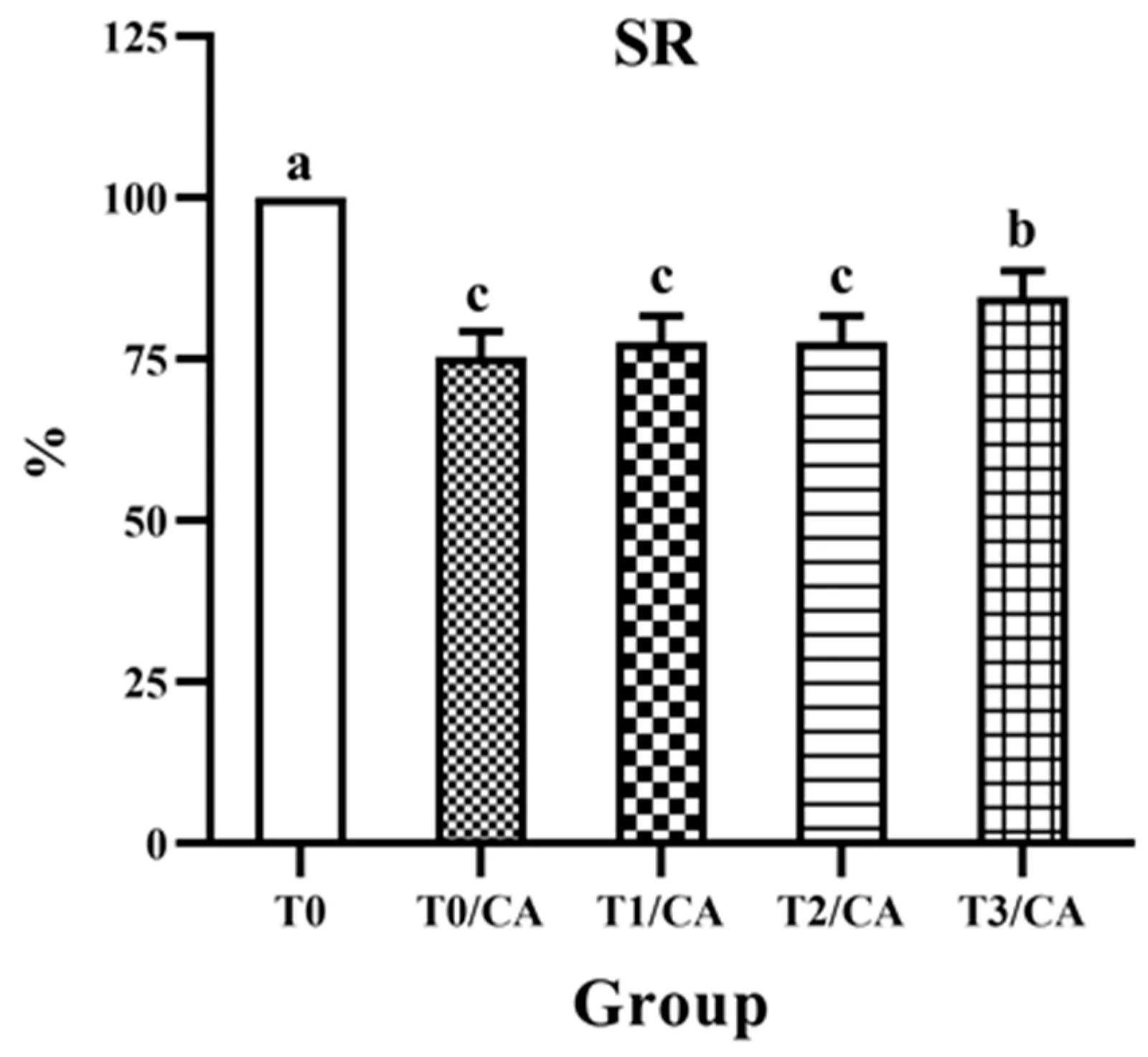
Effect of taurine on survival rate (SR) of *Eriocheir sinensis* exposed to 35.00 mmol/L alkalinity water for 96 h (*n* = 3). Statistical significance (*p* < 0.05) is indicated with different lowercase letters for the same concentration at different time points.

As indicated in Figure 1, the survival rate of the crab was significantly reduced when exposed to 35.00 mmol/L alkalinity stress for 96 h compared with the T0 group. However, injection of taurine increased the survival rate of the crab, which reached the highest value in the T3/CA group and was significantly higher than that in the T0/CA group.

### 3.2. Physiology and Biochemistry

**Figure 2:**
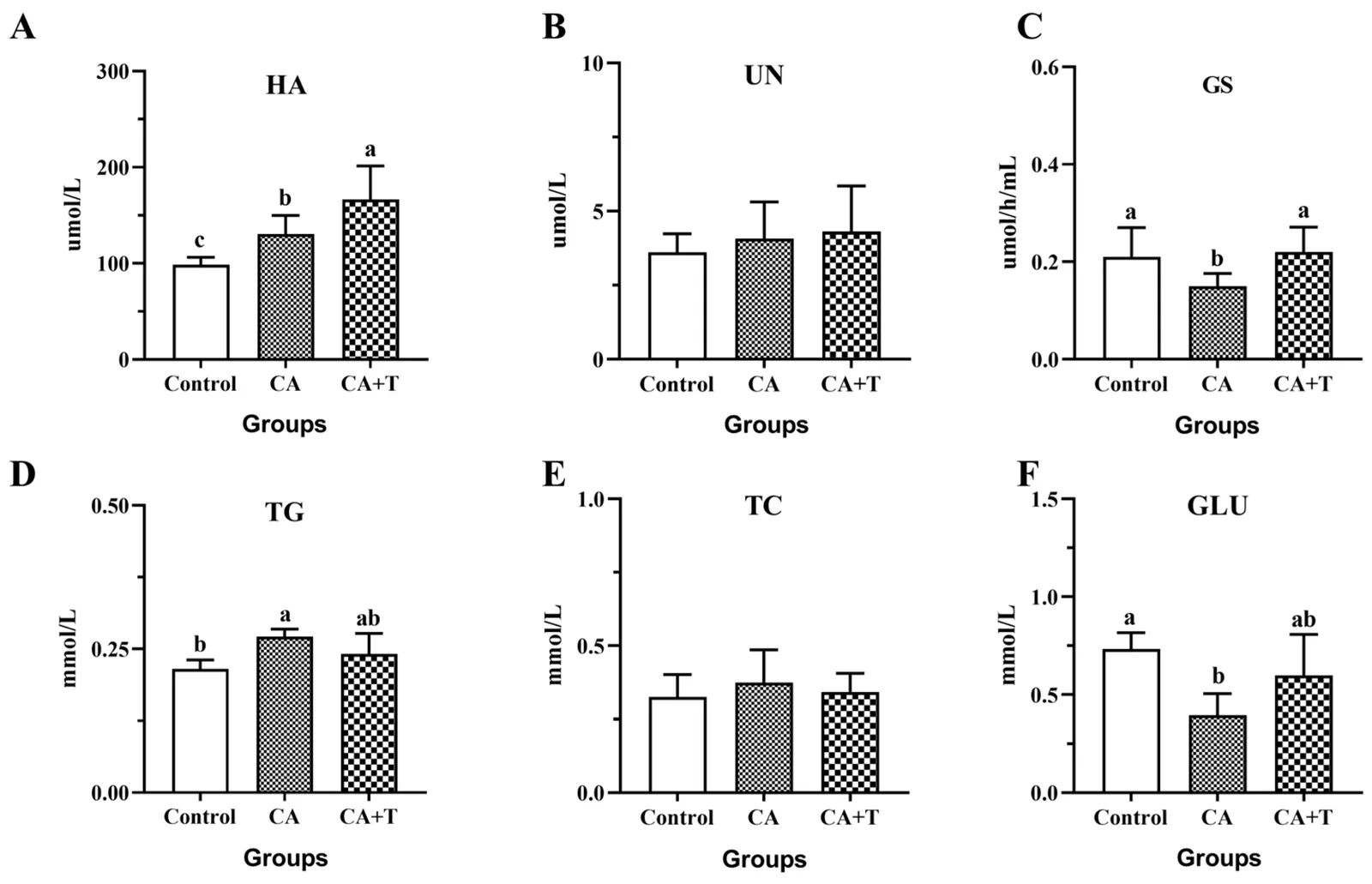
Effect of taurine on physiology and biochemistry of hemolymph of *Eriocheir sinensis* exposed to 17.50 mmol/L alkalinity water for 96 h. (**A**) HA, hemolymph ammonia; (**B**) UN, urea nitrogen; (**C**) GS, glutamine synthetase; (**D**) TG, triglyceride; (**E**) TC, total cholesterol; (**F**) GLU, glucose (*n* = 6). Statistical significance (*p* < 0.05) is indicated with different lowercase letters for the same concentration at different time points.

Enhanced by taurine injection, the ammonia content in hemolymph significantly increased and exceeded that of both the control and alkalinity treatment groups (*p* < 0.05), as depicted in Figure 2. Following alkalinity stress, there was an increase in urea nitrogen content in the hemolymph. Notably, post-taurine injection, the urea nitrogen content in the hemolymph was higher than that in alkalinity group, although no significant difference was observed between the control and alkalinity treatment groups (*p* > 0.05). Alkalinity stress significantly increased the triglyceride content in crab hemolymph (*p* < 0.05), whereas taurine supplementation reduced these elevated triglyceride levels (*p* > 0.05). In contrast, hemolymph glucose concentration was markedly decreased under alkalinity stress, and taurine treatment triggered an increase in glucose content (*p* > 0.05). No significant difference was observed in total cholesterol content across all experimental groups (*p* > 0.05).

### 3.3. Antioxidant Capacity

**Figure 3:**
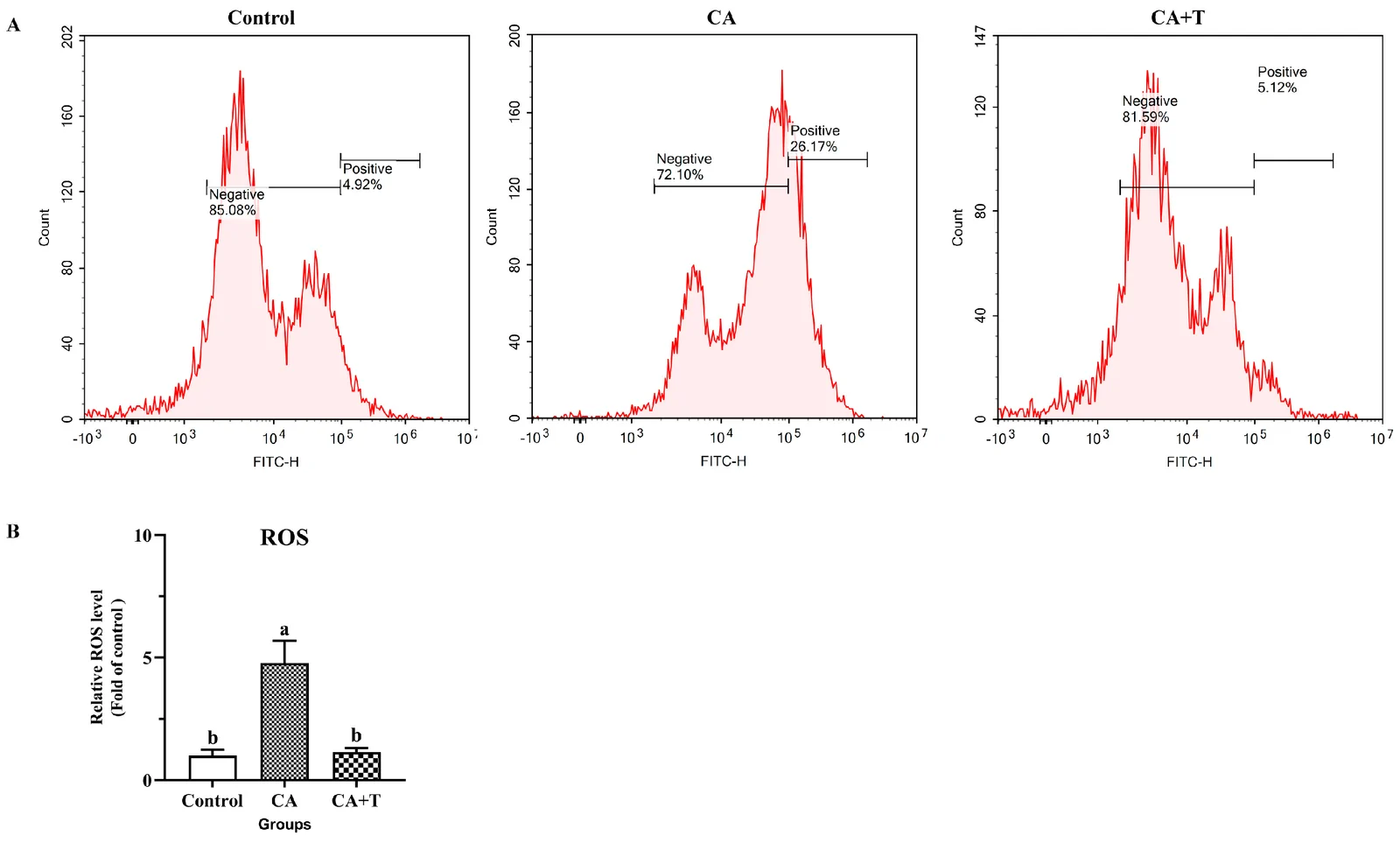
Effect of taurine on the content of ROS in hemolymph of *Eriocheir sinensis* exposed to 17.50 mmol/L alkalinity water for 96 h (*n* = 3). (**A**) Representative flow–cytometry histograms showing the proportions of ROS–positive hemocytes in the Control, CA, and CA + T groups. (**B**) Quantitative analysis of relative ROS levels (fold change relative to the control group). Statistical significance (*p* < 0.05) is indicated with different lowercase letters for the same concentration at different time points.

To investigate whether alkalinity stress induces oxidative stress in crab and whether taurine can modulate alkalinity-induced oxidative stress, flow cytometry was employed to detect the levels of reactive oxygen species (ROS) in hemolymph. The results, as shown in Figure 3, revealed that compared to the control group, the ROS levels significantly increased in the CA group (*p* < 0.05), while taurine markedly reduced the ROS levels in hemolymph (*p* < 0.05).

**Figure 4:**
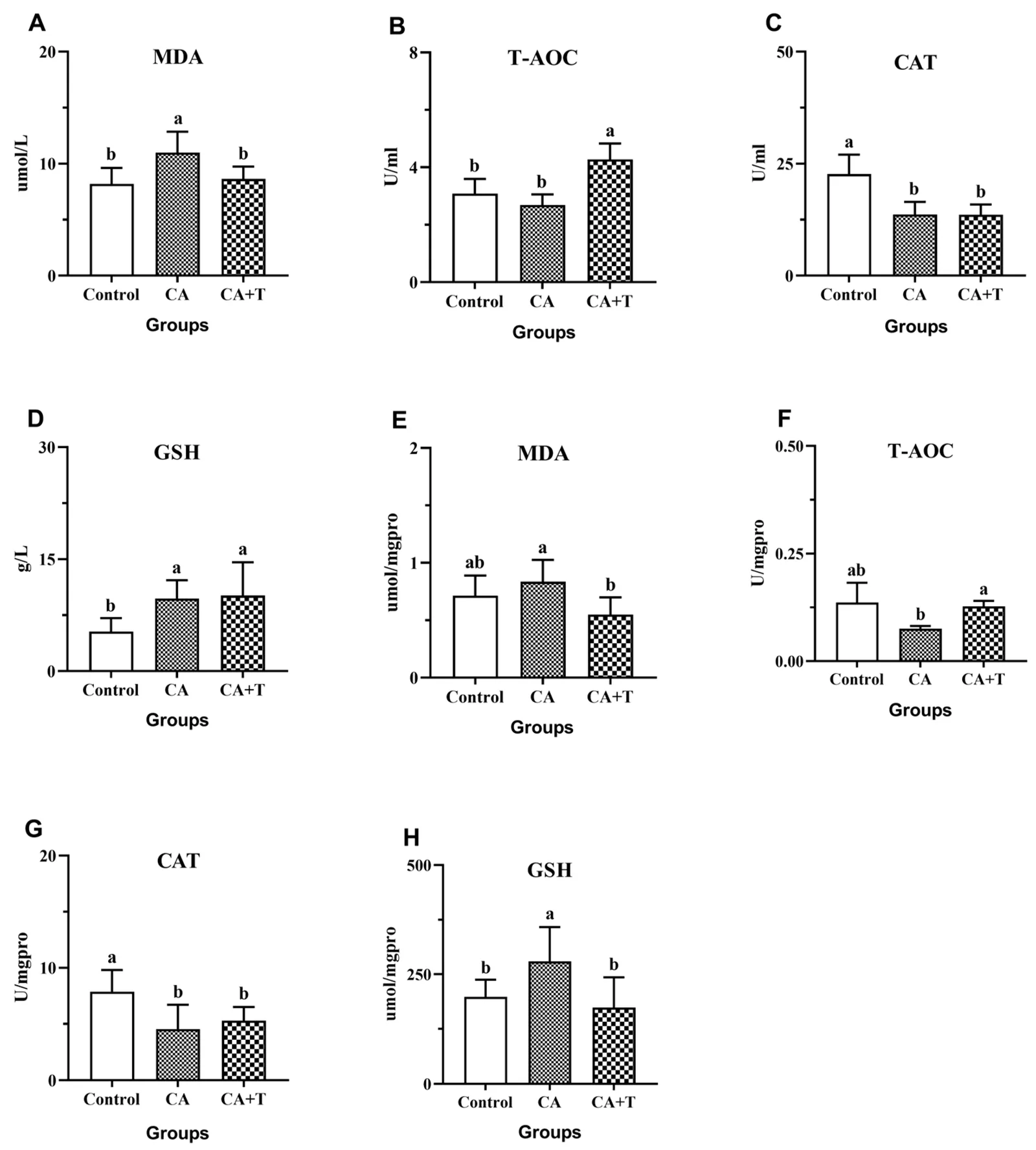
Effect of taurine on antioxidant capacity of *Eriocheir sinensis* exposed to 17.50 mmol/L alkalinity water for 96 h. Panels (**A**–**D**) represent Malondialdehyde level, total antioxidant capacity, catalase activity, and glutathione level in the hemolymph, respectively. Panels (**E**–**H**) depict the corresponding measures in the gills (*n* = 6). Statistical significance (*p* < 0.05) is indicated with different lowercase letters for the same concentration at different time points.

Figure 4 illustrated that under alkalinity stress conditions, both hemolymph and gill tissues exhibited a significant increase in malondialdehyde (MDA) content (*p* < 0.05). However, compared to the CA group, taurine injection significantly reduced the MDA content in both hemolymph and gill tissues (*p* < 0.05), with no significant difference observed compared to the control group (*p* > 0.05). Furthermore, alkalinity stress resulted in a significant decrease in T-AOC in both hemolymph and gill tissues, while taurine injection significantly increased the activity of T-AOC in hemolymph and gill tissues after alkalinity stress (*p* < 0.05). Alkalinity stress significantly reduced the activity of CAT in both hemolymph and gill tissues, with taurine injection showing no significant impact (*p* > 0.05). Under alkalinity stress conditions, the content of GSH significantly increased in both gill and hemolymph tissues. Although taurine injection did not significantly affect the GSH content in hemolymph tissues (*p* > 0.05), it significantly reduced the GSH content in gill tissues compared to the alkalinity stress group (*p* < 0.05).

### 3.4. Flow Cytometry Analysis

**Figure 5:**
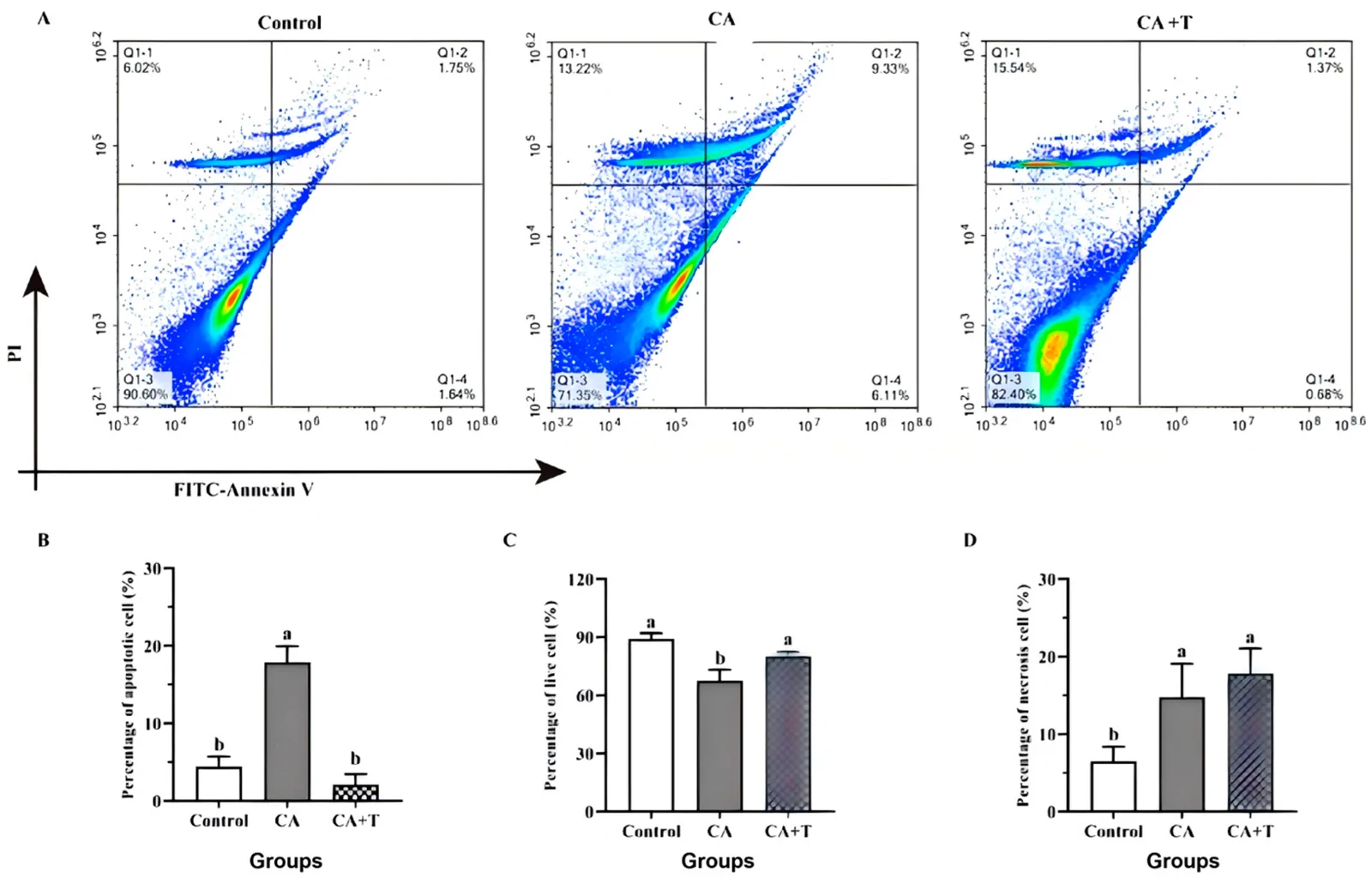
Effect of taurine on the apoptosis of blood lymphocytes of *Eriocheir sinensis* exposed to 17.50 mmol/L alkalinity water for 96 h. (**A**) The quantitative results of flow cytometry in blood lymphocytes. Q1–2 and Q1–4 indicates apoptotic cells, Q1–3 shows live cells. Density dot–plot of hemocytes. The color gradient represents cell density: blue indicates low cell density, and green–yellow–red indicates progressively higher cell density, with red representing the region of maximum cell abundance. (**B**–**D**) The ratios of apoptotic and live cells in flow cytometry, *n* = 3. Statistical significance (*p* < 0.05) is indicated with different lowercase letters for the same concentration at different time points.

As shown in Figure 5A, both alkalinity stress and taurine injection significantly affect apoptosis of hemolymph cells in *E. sinensis*. The percentage of viable cells was 90.60% in the control group, which decreased to 71.35% under alkalinity stress. Meanwhile, the proportion of apoptotic cells was elevated from 1.75% to 9.33%. Taurine supplementation reversed this change, with the percentage of viable cells recovering to 82.40% and late-apoptotic cells dropping to 1.37%. In Figure 5B–D, it can be observed that alkalinity stress significantly increased the proportion of apoptotic and necrotic hemolymph cells while decreasing the proportion of viable cells, from 3.39% to 15.44%, 6.02% to 13.22%, and 90.60% to 71.35%, respectively. However, taurine injection significantly reduced apoptosis and increased the proportion of viable cells in hemolymph, reaching 2.05% and 82.4%, respectively. Nevertheless, compared to the CA group, taurine had no significant effect on the rate of cell necrosis (*p* > 0.05).

### 3.5. Apoptosis-Related Gene Expression

**Figure 6:**
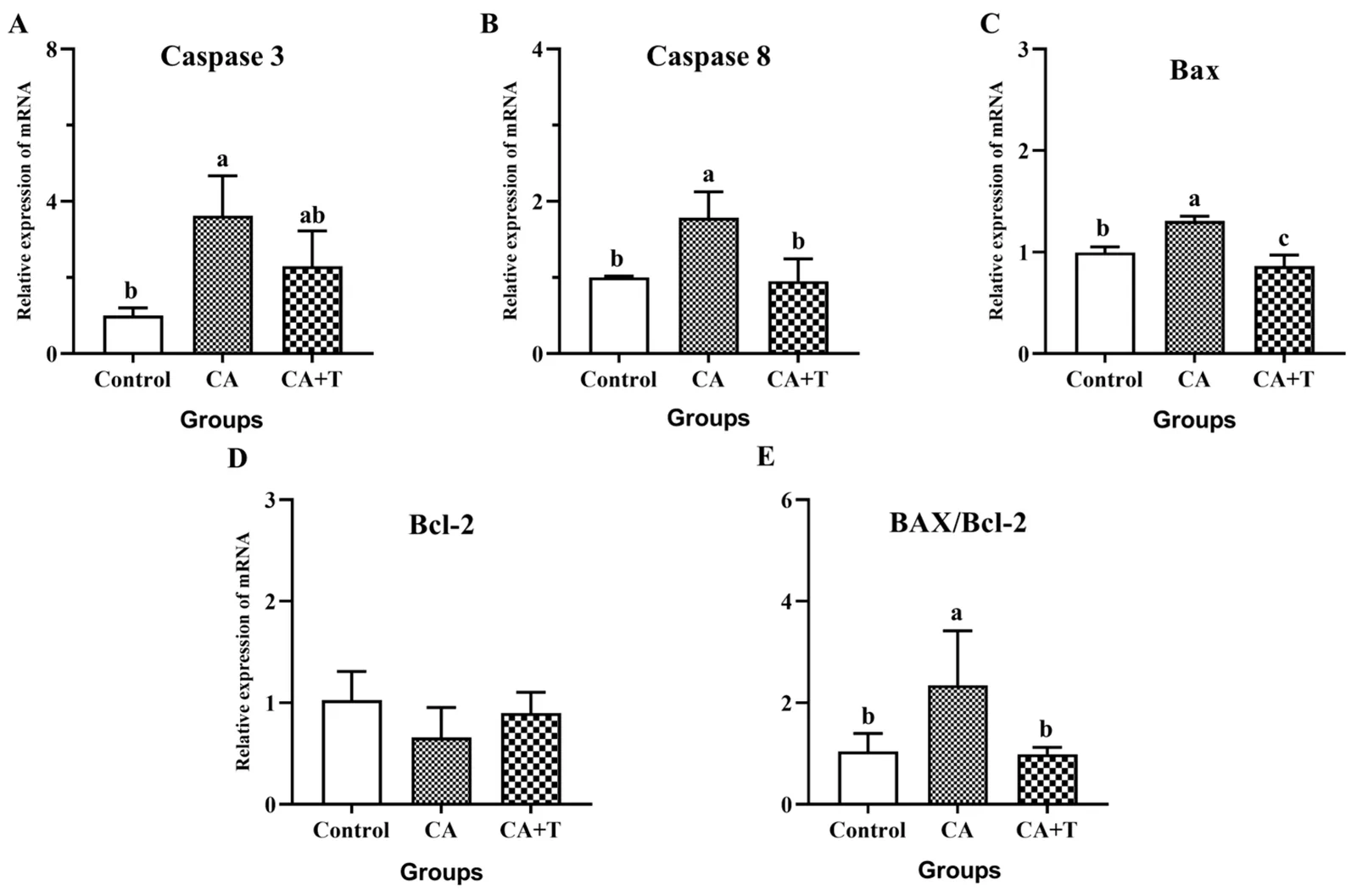
The effect of taurine on the expression of apoptosis-related genes in the gills of *Eriocheir sinensis* exposed to 17.50 mmol/L alkalinity water for 96 h (*n* = 6). (**A**) The relative mRNA expression of *Caspase 3*. (**B**) The relative mRNA expression of *Caspase 8*. (**C**) The relative mRNA expression of *Bax*. (**D**) The relative mRNA expression of *Bcl-2*. (**E**) The ratio of Bax to Bcl-2 mRNA expression level. Statistical significance (*p* < 0.05) is indicated with different lowercase letters for the same concentration at different time points.

According to Figure 6, it is observed that alkalinity stress leads to an upregulation in the relative expression of the *Bax* gene in the gills. However, taurine injection significantly reduced the relative expression of the *Bax* gene to notably lower than that of the control group (*p* < 0.05). Additionally, alkalinity stress exerted a certain inhibitory effect on the relative expression of the *Bcl-2* gene, although the difference compared to the control group was not significant. Furthermore, it can be observed from the figure that the *Bax/Bcl-2* ratio significantly increased after alkalinity stress (*p* < 0.05), while taurine injection significantly reduced the increase in the *Bax/Bcl-2* ratio (*p* < 0.05). Moreover, the relative expression levels of *Caspase 3* and *Caspase 8* genes in the gills significantly increased after alkalinity stress (*p* < 0.05), but taurine significantly downregulated the relative expression levels of *Caspase 8* genes (*p* < 0.05).

### 3.6. MAPK Signaling Pathway

**Figure 7:**
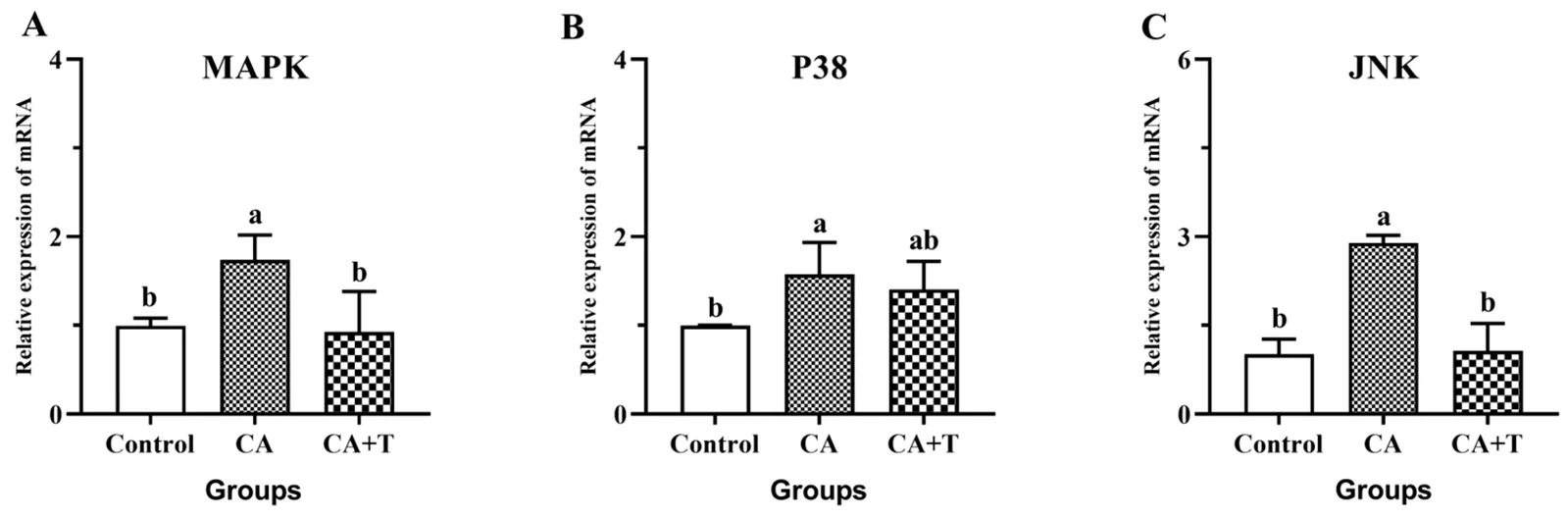
The effect of taurine on the expression of MAPK signaling pathway-related genes in the gills of *Eriocheir sinensis* exposed to 17.50 mmol/L alkalinity water for 96 h (*n* = 6). (**A**) The relative mRNA expression of MAPK (**B**) The relative mRNA expression of P38. (**C**) The relative mRNA expression of JNK. Statistical significance (*p* < 0.05) is indicated with different lowercase letters for the same concentration at different time points.

According to Figure 7, alkalinity stress induced a significant upregulation in the relative expression levels of *MAPK* genes in the gills of the crab. However, the injection of taurine significantly inhibited the upregulation of *MAPK* genes in the gills of Chinese mitten crab before alkalinity stress. The expression of *JNK* gene exhibited a comparable expression trend. Alkalinity stress also markedly elevated the transcript abundance of the *p38* gene, and taurine injection reduced its expression in a non-significant manner. It is noteworthy that there was no significant difference in the expression levels of the *P38* gene between the CA + T group and the CA group (*p* > 0.05).

### 3.7. Molecular Docking Results

**Figure 8:**
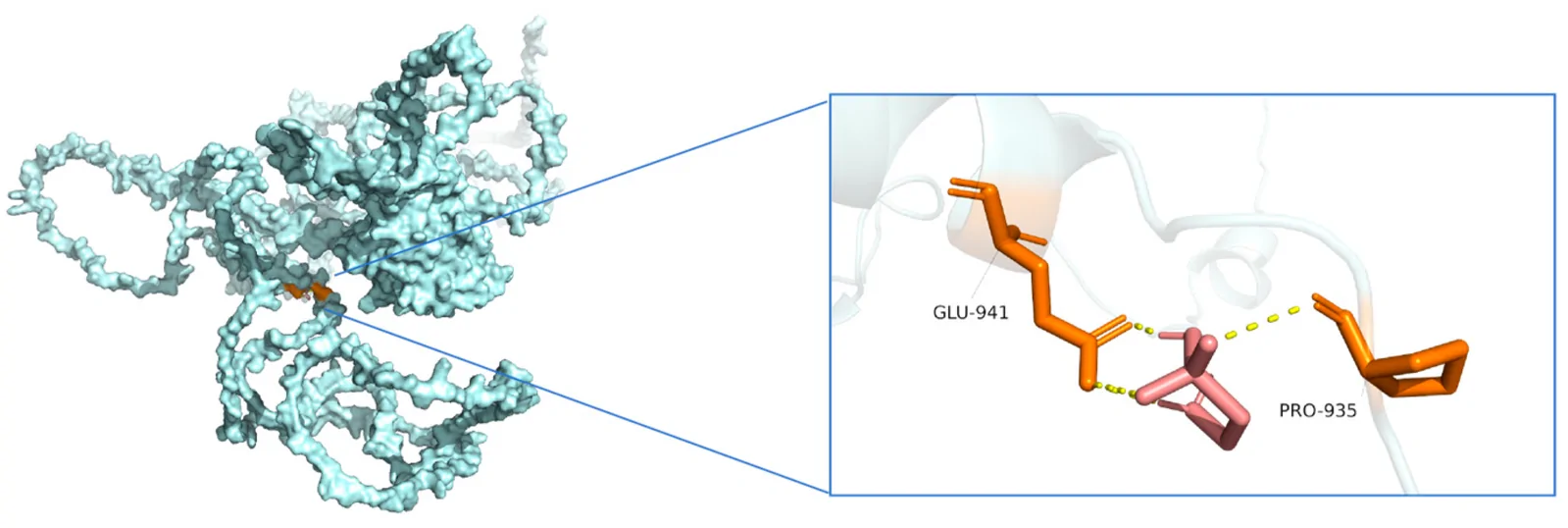
Molecular docking results of taurine molecule and *MAPK* protein. The yellow dashed lines represent hydrogen–bond interactions.

To investigate whether taurine can bind to MAPK protein, molecular docking of taurine with MAPK was performed (Figure 8). The results predicted that the ligand docking would occur with amino acid glutamic (Glu) 941 of the receptor protein with hydrogen bonds. Visualization using Pymol (version 2.6.0) revealed that the ligand was potentially docking with amino acid glutamic (Glu) 941 of the receptor protein through four hydrogen bonds, and there is a possibility of hydrogen bond formation with amino acid proline (Pro) 935 through one hydrogen bond. It was therefore hypothesized that taurine may have a potential binding affinity for MAPK protein, though further experiments are required to verify this prediction.

## 4. Discussion

High saline–alkaline water, while it may not directly result in the mortality of certain fish and crustaceans, does lead to a negative effect on aquaculture animals, ultimately affecting production efficiency [26,27]. In recent years, researchers attempted to promote the growth of aquatic animals and enhance their antioxidant and immune capabilities through nutritional regulation. However, there is limited research on feed formulations for aquatic animals in saline–alkaline water conditions, particularly with a focus on *E. sinensis*. Taurine is known for its role in enhancing the antioxidant capacity and growth performance of aquatic animals, offering potential in mitigating alkalinity stress. In this study, we investigated whether taurine could alleviate the damage caused by alkalinity stress and elucidate the underlying mechanisms, aiming to provide theoretical evidence for the application of taurine as a functional feed additive in commercial saline–alkali aquaculture. The results showed that taurine could reduce the ROS content in hemolymph, alleviate alkalinity-induced oxidative stress, and decreased cell apoptosis by inhibiting the expression of *MAPK* and *Caspase 3* mRNA and decreasing the Bax/Bcl-2 ratio.

Alkalinity refers to the proton-binding or acid-neutralizing capacity of water, originating from dissolved proton-accepting constituents, primarily HCO_3_^−^, CO_3_^2−^, OH^−^ and H_4_BO_4_^−^, with bicarbonate being the dominant fraction. Optimal alkalinity can alleviate heavy metal toxicity, modulate CO_2_ dynamics and stabilize water pH, whereas high alkalinity produces toxic impacts on aquatic fauna [28]. Previous investigations indicated that alkalinity stress disturbs respiratory metabolism, ammonia homeostasis, antioxidant defense, immune function, apoptosis and autophagy [29]. Dysregulated ammonia metabolism can result in ammonia buildup, thereby inducing physiological injury [4]. Ammonia is produced from the breakdown metabolism of proteins in the fish’s body and is excreted from the blood through the gills [30]. Impaired ammonia excretion can lead to excessive accumulation of ammonia in the body, resulting in physiological responses such as oxidative stress, inflammatory reactions, cell apoptosis, tissue damage, and central nervous system disorders [31,32]. In this study, it was found that alkalinity stress could cause accumulation of ammonia in the hemolymph and reduce the activity of GS. The reason for this may be that the decreased content of proton (H^+^) in the water body prevents ammonia (NH_3_) from capturing proton (H^+^) to form ammonium ion (NH_4_^+^) for excretion, thus leading to the accumulation of ammonia in the body [33]. Taurine is an effective immunomodulatory and anti-inflammatory factor associated with various health benefits, including tissue repair, antioxidation, immune regulation, alleviation of metal toxicity, osmoregulation, and neuroregulation [29,30,31,32,33]. Chepkova et al. [34] reported that taurine may alleviate hyperammonemia in mice by improving mitochondrial function, regulating the activity of GS, and reducing the accumulation of ammonia. Ren et al. [35] also found that taurine injection could reduce blood ammonia levels and alleviate the adverse effects of hyperammonemia on crucian carp by regulating the activity of GS and the ornithine–urea cycle. However, under the conditions of this experiment, although taurine injection increased the activity of GS and urea nitrogen content in the hemolymph, ammonia levels also significantly increased compared to the CA group, which may be related to the dosage of taurine injection and the species. Furthermore, we speculate that during the metabolism of taurine in *E. sinensis*, amino groups may be produced, leading to an increase in blood ammonia levels in the hemolymph of *E. sinensis*, which may be related to the presence of amino groups in their own structure.

ROS are important by-products of fish metabolism. Changes in water conditions, such as temperature, pH, alkalinity, and salinity, may stimulate ROS generation, disrupting the dynamic balance between ROS generation and clearance [24,36]. Normally, fish can defend themselves against oxidative stress with the help of their intrinsic antioxidant defense system. The core enzymes in these defense systems, such as SOD and CAT, effectively eliminate harmful ROS derivatives. SOD converts superoxide anions (O_2_^−^) into hydrogen peroxide (H_2_O_2_), which is then catalyzed by CAT and GSH in a synergistic manner, ultimately converting it into water (H_2_O) and gas oxygen (O_2_). However, once stress exceeds the regulatory capacity of fish, excess ROS may attack phospholipids, membrane receptors, and enzyme-associated polyunsaturated fatty acids on biomembranes, leading to lipid peroxidation and the generation of lipid peroxidation products such as MDA [24]. The present study found that alkalinity stress significantly increased the activity of SOD while simultaneously significantly reducing the activity of CAT, resulting in a significant increase in ROS levels. Additionally, the content of MDA in the hemolymph and gills also increased, although not significantly in the gills. These results suggested that the increase in ROS induced by alkalinity stress may be primarily related to the inhibition of CAT activity. However, it is worth noting that taurine did not increase the activity of CAT, yet it successfully reduced the levels of ROS and MDA. This may be related to the antioxidant effect of taurine, which can scavenge free radicals, enhance the body’s antioxidant capacity, and protect the body from oxidative damage [37]. As a comprehensive indicator for evaluating the functionality of the antioxidant enzyme system, T-AOC can reflect the overall antioxidant capacity of non-enzymatic and enzymatic systems. In this study, it was found that taurine injection significantly increased the T-AOC activity in both the hemolymph and gills, which not only demonstrated the ability of taurine to enhance the antioxidant capacity of crabs but also may be one of the potential reasons for the reduction in ROS and MDA levels. Similar findings has been reported in *Monopterus albus*, indicating that taurine could increase the T-AOC activity of the liver, reduce MDA levels, and thereby improve the oxidative stress induced by oxidized fish oil in the diet [38]. These findings reveal that taurine relieves alkalinity-induced oxidative stress, which provides a basis for developing functional feeds for *E. sinensis* reared in carbonate saline–alkaline water.

The MAPK pathway is a key cellular signaling pathway, primarily composed of extracellular signal-regulated kinase (ERK), c-Jun N-terminal kinase (JNK), and p38 kinase [39,40]. In response to various environmental stresses, the MAPK pathway phosphorylates serine/threonine dual sites, activating multiple core transcription factors, thereby regulating numerous cellular physiological processes such as cell growth, differentiation, apoptosis, and autophagy [41]. Among these, JNK and p38 play significant roles in cellular stress, apoptosis, and inflammatory responses, contributing crucially to the maintenance of cellular homeostasis. The activation of the MAPK pathway induced by ROS has been demonstrated to mediate cell apoptosis. It was found that acetochlor could lead to excessive accumulation of ROS and activation of the MAPK pathway, which could induce apoptosis and necrosis in grass carp (*Ctenopharyngodon idellus*) kidney cells [19]. Zhou et al. [42], in a study on rainbow trout (*Oncorhynchus mykiss*) gonadal RTG-2 cells, found that 2, 2′, 4, 4′-tetrabromodiphenyl ether could induce apoptosis by stimulating intracellular ROS production to increase P38 MAPK gene expression. Similar results were also found in the present study. Under carbonate alkaline water conditions, the mRNA expression of *P38*, *JNK*, and *MAPK* in the gills of crabs significantly increased with the excessive production of ROS. Additionally, apoptotic regulatory factors, such as *Caspase3*, *Caspase8*, *Bax*, and the *Bax/Bcl2* ratio, also showed significant elevation. These results indicated that the generation of ROS mediates the activation of the MAPK pathway, promoting cell apoptosis. Flow cytometry results also confirmed this. However, after injection of taurine, cell apoptosis was inhibited. Taurine has been proven to be an effective endogenous antioxidant that can protect HK-2 cells from LDL-induced cytotoxicity by inhibiting ROS-mediated cell apoptosis [43]. This may also be the reason for taurine’s inhibition of alkaline stress-induced cell apoptosis. Furthermore, based on the molecular docking results, it was found that taurine could dock with MAPK protein through hydrogen bonds. Such binding interaction is likely to suppress the MAPK signaling cascade and thereby alleviate cell apoptosis, which is consistent with previously reported cytoprotective mechanisms of taurine against stress-induced injury [44].

## 5. Conclusions

In summary, high alkalinity stress can cause an increase in hemolymph ammonia levels, trigger oxidative stress, and cause apoptosis through the ROS/MAPK signaling pathway, while taurine can exert a protective role, promoting maintenance of a physiological state of redox balance and inhibiting the occurrence of apoptosis by suppressing the MAPK signaling pathway.

## Figures and Tables

**Table 1:** Primer pair sequences for RT-qPCR in *Eriocheir sinensis*.

Gene Names	Primer Sequence Forward (5′-3′)	Primer Sequence Reverse (3′-5′)
*P38*	CAGAAGTGCGGGAGGTGGTG	GTCTTCACGCCCTCCACCAC
*JNK*	GATACGGCGAGGACTCAGG	CTATGCCGCTCCTGAGTCC
*MAPK*	CATCACCAGGACGTGAACGA	CCACTCCCAGCTTGTCCATT
*Caspase 3*	AGTTTTGGGGAGGTGC	TCAGGTTCCGTGGGTT
*Caspase 8*	ACGGTGAAATCTGCCTT	AATCTACGCCAACACGC
*Bax*	GGAGCAGCAGTGGAGTTGGTAAT	GTGGTAGGTGTGGTTGTGTTGGA
*Bcl-2*	GCTCAGGGCAGCGTGT	GCAACCCAGACTCAATCAA
*S27*	CCCCCAAGAAGATCAAGCACA	CAGATGGCAGCGACCACAGTA
*β-actin*	TCGTGCGAGACATCAAGGAAA	AGGAAGGAAGGCTGGAAGAGTG

## Data Availability

The raw data supporting the conclusions of this article will be made available by the authors on request.
